# Lecithin-Polysaccharide Self-Assembled Microspheres for Resveratrol Delivery

**DOI:** 10.3390/antiox11091666

**Published:** 2022-08-26

**Authors:** Lei Wang, Congting Lai, Dong Li, Zisheng Luo, Lingling Liu, Yunbin Jiang, Li Li

**Affiliations:** 1 Key Laboratory of Agro-Products Postharvest Handling, Ministry of Agriculture and Rural Affairs, College of Biosystems Engineering and Food Science, Zhejiang University, Hangzhou 310058, China; 2 Ningbo Research Institute, Zhejiang University, Ningbo 315100, China; 3 National-Local Joint Engineering Laboratory of Intelligent Food Technology and Equipment, Zhejiang University, Hangzhou 310058, China; 4 Zhejiang Key Laboratory for Agro-Food Processing, Zhejiang Engineering Laboratory of Food Technology and Equipment, Zhejiang University, Hangzhou 310058, China; 5 School of Biological and Chemical Engineering, Zhejiang University of Science and Technology, Hangzhou 310023, China; 6 Tianjin Gasin-DH Preservation Technologies Co., Ltd., Tianjin 300403, China

**Keywords:** resveratrol, encapsulation, soybean lecithin, polysaccharide, microspheres, stability

## Abstract

Poor water solubility and low chemical stability, seriously limit the efficient bioavailability of resveratrol. Here, we propose encapsulating resveratrol in lecithin-polysaccharide self-assembled microspheres (LPSM). An LPSM was designed with a lecithin core, and alginate-carboxymethyl chitosan biolayer shell. The LPSM had a spherical shape with 12.171 ± 0.960 μm of particle size and −30.86 ± 1.37 mV of zeta potential. The introduce of lecithin remarkably increased the encapsulation efficiency of resveratrol to 92.78 ± 0.82%. The LPSM elevated the antioxidant capacity and ultraviolet resistance of resveratrol. Moreover, LPSM inhibited release in a simulated gastric environment, promoted sustained release in simulated intestinal environment, and elevated the bioavailability of resveratrol during in vitro simulated digestion. Results indicate that LPSM is promising as a carrier for resveratrol delivery to enhance stability and bioaccessibility.

## 1. Introduction

Resveratrol (trans-3,5,4′-trihydroxy-stilbene) is a polyphenolic compound present in mulberries, peanuts, and grapes, among others [1,2,3]. The potential benefits of resveratrol for health have been widely proven by numerous studies based on in vitro and in vivo investigations, including anti-obesity [2], anti-inflammatory [3], anti-carcinogenic [4], heart-protective [5] and brain benefit effects [6]. It is regarded as a multifunctional bioactive agent in food and pharmaceuticals with considerable interest. Nevertheless, its poor water solubility, low chemical stability and bioavailability, seriously limit the application of resveratrol. The intrinsic resveratrol compound is slightly soluble in water, only 0.021–0.030 mg mL^−1^, impeding the incorporation of resveratrol into aqueous-based food [7]. More importantly, resveratrol is prone to chemical degradation due to exogenous environment stress stimuli, including temperature, ultraviolet light, pH, and enzymes [8,9]. Resveratrol as food antioxidant or food additive also tends to undergo rapid and extensive degradation during ingestion, reducing its bioavailability and bioaccessibility [10].

Encapsulation of food-grade resveratrol has gained increasing attention to overcome the challenges mentioned above [11]. Application of bioactive compound loading with encapsulating agents allows the formation of a capsule that protects the core from degradation induced by external environment stimuli [12]. Biological molecules, including proteins, polysaccharides, and lipids, are used as food-grade carriers for the fabrication of capsules [13]. Leena et al. [14] developed electro-spinning zein nanofibers with an encapsulation efficiency for resveratrol up to 96.9%, effectively protecting resveratrol when exposed to simulated gastric fluid. Jayan et al. [15] used an electro-spraying technique to encapsulate resveratrol in zein for the improvement of bioavailability. Sanna et al. [16] prepared novel cationic chitosan-coated and anionic alginate-coated poly (D,L-latctide-co-glycolide) nanoparticles loaded with resveratrol, which provided significant protection against degradation and transisoformation caused by light exposure. The electro-spinning encapsulation of resveratrol into fibers provided localized delivery and improved the apoptotic effect on K562 cancer cells [17].

Lipid-based encapsulation, including emulsion, solid lipid nanoparticles, and liposomes has unique advantages compared to the protein-based and polysaccharide-based encapsulation, resulted from higher solubilities of resveratrol. Bryla et al. [18] reported soybean lecithin exhibited great stability for the formation of liposomes. Huang et al. [19] used liposome encapsulation as a strategy for the delivery of resveratrol with improved encapsulation efficiency. A lipid-core nanocapsule containing resveratrol was developed by Coradini et al. [20] for elevated photostability and controlled release. Balanc et al. [21] developed a novel resveratrol delivery system based on alginate-sucrose and alginate-chitosan microbeads containing liposomes, focusing on the composition of the polysaccharide layer. However, it is still unknown how the existence of the lipid core influenced the delivery characteristics of polysaccharide-encapsulated resveratrol.

**Figure 1:**
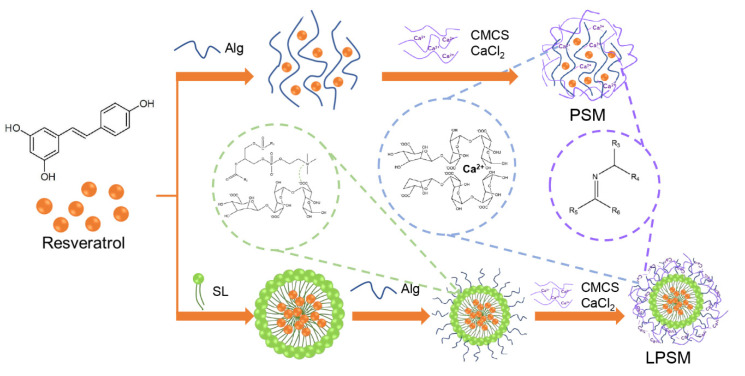
Preparation of polysaccharide self-assembled microspheres (PSM) and lecithin-polysaccharide self-assembled microspheres (LPSM). Alg, alginate; SL, soybean lecithin; CMCS, carboxymethyl chitosan.

In the present study, a lipid core was introduced into polysaccharide self-assembled microspheres (PSM) to develop lecithin-polysaccharide self-assembled microspheres (LPSM, Figure 1) for improvement of resveratrol delivery properties. Subsequently, scanning electron microscopy and Fourier infrared spectroscopy were used to analyze the structure of microspheres. Then, the particle size, zeta potential, and encapsulation efficiency (EE) were measured. Antioxidant capability, ultraviolet resistance, and in vitro release were determined to evaluate the effectiveness of the two encapsulation methods. The purpose of the present study was to develop a promising method to enhance the stability and bioaccessibility of resveratrol.

## 2. Materials and Methods

Preparation of microspheres. All experimental operations were performed in dark conditions and carried out on three independent batches as three biological replications. Resveratrol (0.05 g, 99%, bought from Aladdin, pre-dissolved in 1 mL ethanol) was dissolved in deionized water to 100 mL. An equal volume of 3% sodium alginate (analytical reagent, bought from Aladdin) solution was added, and stirred for 15 min, then solid microspheres were collected in the collection solution, containing 0.5% carboxymethyl chitosan (CMCS, biochemical regent, bought from Aladdin) and 2% calcium chloride (analytical reagent, bought from Aladdin) for 10 min. Polysaccharide self-assembled microspheres (PSM) were obtained after freezing under vacuum for 24 h. Soybean lecithin (2 g, 98%, bought from Aladdin) and 0.05 g of resveratrol (pre-dissolved in 1 mL ethanol) were dissolved in deionized water to 100 mL and stirred at the speed of 800 rpm for 50 min. Then, the liposome suspension was added into an equal volume of 3% sodium alginate solution and stirred for 15 min. Solid microspheres were collected in a collection solution containing 0.5% CMCS and 2% calcium chloride, for 10 min. Lecithin-polysaccharide self-assembled microspheres (LPSM) were obtained after freezing under vacuum for 24 h.

Fourier transform infrared spectroscopy (FTIR) analysis. FTIR was used to measure structural changes of PSM and LPSM. FTIR spectra were recorded using an FT-IR spectrophotometer (FTS 135, Bio-Rad, Hercules, CA, USA) at a resolution of 2 cm^−1^ with a scanning wavelength of 4000–400 cm^−1^.

Scanning electron microscope (SEM) images. The morphology of the PSM and LPSM were examined by SEM (SEM, S-4800, Hitachi, Tokyo, Japan). Freeze-dried powders glued in two squares on round metal. Vacuum coating was carried out in a vacuum instrument with 2 kV to form a gold film of about 10 nm, then the samples were examined by SEM.

Particle size and potential of microspheres. According to the methods of Chaudhari et al. [22], the freeze-dried microsphere samples were diluted with water to a certain concentration and a multi-angle particle size analyzer was used to determine nanoparticle size and zeta potential.

Encapsulation efficiency (EE). Encapsulation efficiency was determined by the extraction of total resveratrol and free resveratrol. A quantity of 10 mg of nanoparticles loaded with resveratrol was dissolved in 10 mL of 0.01 mol L^−1^ sodium citrate solution and then vortexed vigorously for 10 min at ambient temperature. After centrifugation at 10,000 rpm for 10 min, the supernatant was taken to measure the content of total resveratrol. The resveratrol was determined by high-performance liquid chromatography (HPLC, Agilent technologies 1290 infinity) using a Welch ultimate LP-C18 (4.6 × 250 mm). A 1% formic acid was used as mobile phase A and acetonitrile was used as mobile phase B for gradient elution. The linear gradient program was: 0–56 min, 3–30% B in A; 56–79 min, 24–67% B in A; 79–81 min, 67–100% B in A; 81–85 min, 100–0% B in A. Resveratrol was quantified based on standard curves (y = 17.628x − 5.7081, R^2^ = 0.9999) at 280 nm. Each experiment was carried out in triplicate. The encapsulation efficiency of resveratrol was calculated as follows:

EE = (Total amount of resveratrol-free resveratrol)/(Total amount of resveratrol) × 100% (1)

2,2-diphenyl-1-(2,4,6-trinitrophenyl) hydrazyl (DPPH) radical scavenging activity. DPPH radical scavenging activities of free resveratrol (FR), PSM, and LPSM were measured according to Huang et al. [23] with a slight modification. To determine the antioxidant activity of DPPH of embedded resveratrol microspheres, three solutions were prepared. A volume of 2 mL of 0.2 mmol L^−1^ DPPH (dissolved in ethanol) was mixed with a 2 mL sample and then placed in the dark at 37 ± 0.5 °C for 30 min to obtain a sample at 517 nm. The control was prepared by replacing the sample with 2 mL of anhydrous ethanol. A blank sample was prepared by replacing the DPPH with 2 mL anhydrous ethanol. Each experiment was carried out in triplicate. DPPH scavenging was calculated as follows:

DPPH scavenging = (1 − (A_sample_ − A_control_)/A_blank_) × 100% (2)

where A_sample_ stands for the absorbance of the sample at 517 nm, A_control_ stands for the absorbance of sample at 517 nm, and A_blank_ stands for the absorbance of sample at 517 nm

Ultraviolet (UV) resistance. The retention rates of resveratrol in different forms were determined to evaluate UV resistance. The FR, PSM, and LPSM were placed under UV light for 48 h. The resveratrol content was determined every day. Each experiment was carried out in triplicate. The retention rate was calculated as follows:

Retention rate = *C*_t_/*C*_0_ × 100% (3)

where *C*_0_ stands for the initial content of resveratrol, and *C*_t_ is defined as the content of resveratrol at time t.

In vitro release studies. In vitro release studies of PSM and LPSM were carried out to simulate digestion and absorption in the stomach and intestine according to Huang et al. [18] with slight modifications. To prepare the simulated gastric fluid (SGF), 3.2 g L^−1^ of pepsin was prepared in physiological saline, and the pH of the solution was adjusted to 1.2 using hydrochloric acid. The PSM and LPSM samples in SGF were placed in a thermostatic shaker at 37 ± 0.5 °C, with a continuous shaking at 100 rpm for 2 h. Then, the samples were transferred into simulated intestinal fluid (SIF), which contained 10 g L^−1^ trypsin in phosphate buffer solution (pH 7.4) at 37 ± 0.5 °C, with continuous shaking at 100 rpm for 3 h. At predetermined time intervals (1, 2, 3, 4 and 5 h), the suspensions were centrifuged, and the supernatant was taken to determine the resveratrol concentration by HPLC. The resveratrol was quantified by the areas of peaks based on standard curves. Each experiment was carried out in triplicate. The release rate was calculated as follows.

(4) $$RR_{t}=C_{t}V_{0}+\sum C_{t-1}V$$

where *RR*_t_ stands for the release rate of resveratrol from microspheres, *C*_t_ is the concentration of resveratrol at time t, *C*_t__−1_ is the concentration of resveratrol at time t − 1, *V*_0_ is the total volume of buffer solution, and *V* is the volume of supernatant taken out.

Bioaccessibility was recorded as the final release rate in SIF.

Statistics analysis. The determinations of particle size, Zeta potential, encapsulation efficiency, antioxidant capability, retention rate in response to ultraviolet, and release rate of resveratrol in vitro were carried out in three technical replications. Analysis of variance was conducted using the Duncan test with SPSS software (Version 19.0). All results are presented as means ± standard deviations with significant difference denoted by letters.

## 3. Results and Discussion

### 3.1. Morphology of Resveratrol Microspheres

**Figure 2:**
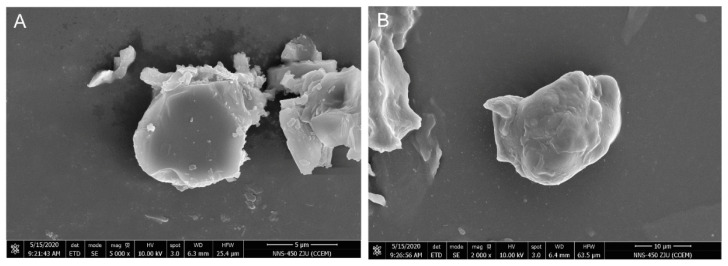
Scanning electron microscopy (SEM) images of polysaccharide self-assembled microspheres (PSM, (**A**)), and lecithin-polysaccharide self-assembled microspheres (LPSM, (**B**)).

Structures of encapsulated resveratrol microspheres are shown in Figure 2. Both PSM and LPSM encapsulations exhibited spherical shapes, which were well suited for embedding resveratrol. Ahmad et al. [24] reported that resveratrol micrographs showed a needle-like structure. In the present study, spherical structures, instead of a resveratrol-like structure, indicated the successful preparation of the two kinds of self-assembled microspheres.

### 3.2. FTIR Analysis

**Figure 3:**
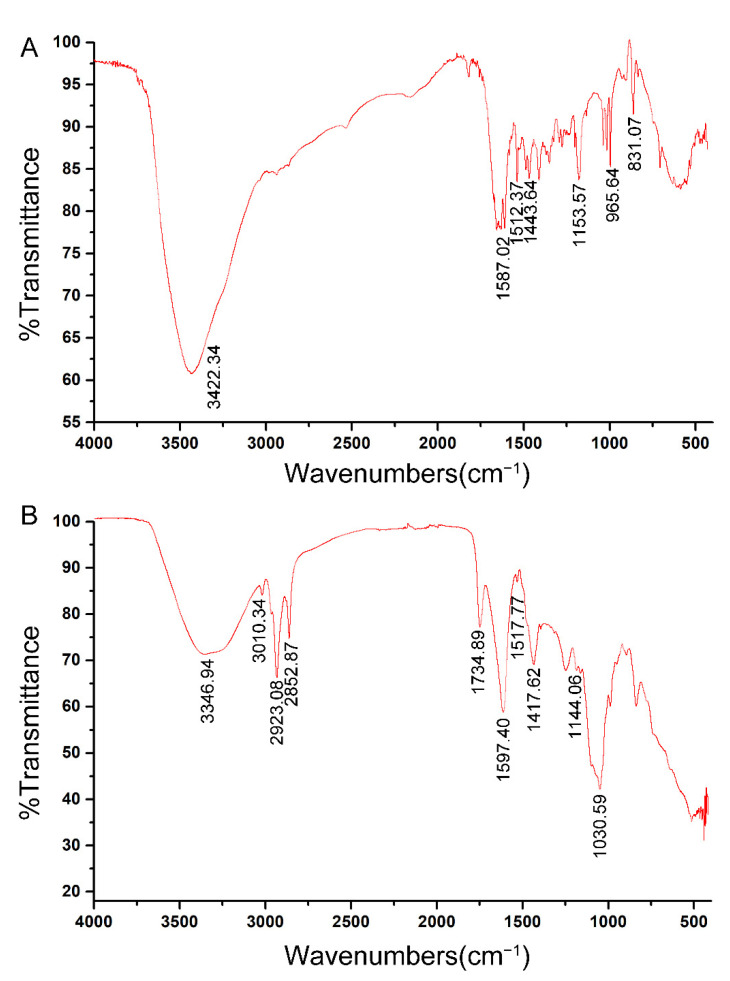
Fourier Transform Infrared (FTIR) spectra of resveratrol loaded in polysaccharide self-assembled microspheres (PSM, (**A**)) and lecithin-polysaccharide self-assembled microspheres (LPSM, (**B**)).

The FTIR spectra of PSM and LPSM loaded resveratrol were shown in Figure 3, and the FTIR spectra of microspheres without resveratrol were shown in Figures S1 and S2. O-H stretching at wavenumbers ranging from 3300 to 3500 cm ^−1^ probably represent the hydroxyl group of polysaccharides and the phenolic hydroxyl group of resveratrol. The peaks at 1587.02 cm^−1^, 1512.37 cm^−1^, and 1443.64 cm^−1^ in PSM, 1597.40 cm^−1^, 1517.77 cm^−1^, and 1417.62 cm^−1^ are the characteristic absorption peaks of the aromatic skeletons in resveratrol, indicating the successful encapsulation of resveratrol. The peaks at 1153.57 cm^−1^ of PSM and 1144.06 cm^−1^ represent the C-O-C stretching of polysaccharides.

Compared to PSM, the peaks at 3010.34 cm^−1^, 2923.08 cm^−1^ and 2852.87 cm^−1^ of LPSM reflect CH_3_ anti-symmetric stretching, CH_3_ symmetrical stretching, and CH_2_ symmetrical stretching, respectively, indicating the presence of soy lecithin (Figure 3B). Due to the addition of soy lecithin, resveratrol was isolated from alginate and carboxymethyl chitosan. The peaks of PSM at the wavenumbers from 1250 cm^−1^ to 1400 cm^−1^ might result from the C-N stretching and CH_2_ swing influenced by encapsulated resveratrol (Figure 3A).

### 3.3. Particle Size and Zeta Potential

The stability of PSM and LPSM microspheres was tested by assessing particle size and zeta potential. As shown in Table 1, the particle sizes of PSM and LPSM microspheres were 6.422 ± 0.721 μm and 12.171 ± 0.960 μm, respectively. This result is in line with that shown in the SEM image. Ahmad et al. [24] prepared nano-encapsulated resveratrol particles with starch from horse chestnut, lotus stem, and water chestnut, and the particle sizes were found to be 419, 797, and 691 nm, respectively. The increase from PSM to LPSM in particle size may be due to the layer-by-layer assembly interaction of polysaccharides and lecithin. The greater size of microspheres might add to the existing soybean lecithin layer, thickening the microspheres.

In the present study, the Zeta potentials of PSM and LPSM, indicating particle stability, were found to be negative, −21.18 ± 1.05 mV and −30.86 ± 1.37 mV, respectively. The negative charge of microspheres could be due to the presence of the carboxyl group of carboxymethyl chitosan. The electrostatic repulsion forces created by the surface charge could decrease the Van der Waals force between particles, avoiding the agglomeration of particles to form bigger particles [11,25]. The higher the zeta potential value, the more difficult for the particles to aggregate [25]. In the present study, it was evident from the higher zeta potential that the stable properties and resistance to aggregation were better maintained by the LPSM compared to PSM. This result may be due to the negative charge of soybean lecithin [18].

### 3.4. Encapsulation Efficiency

EE has been widely used to characterize the encapsulation efficiency of the guest molecule. As shown in Table 1, the EE values for PSM and LPSM were 65.45 ± 0.43% and 92.78 ± 0.82%, respectively, and LPSM exhibited significantly greater encapsulation efficiency than PSM (The HPLC chromatograms were shown in Figures S2–S7). The difference of EE between LPSM and PSM was probably attributed to the lipophicity of resveratrol. The addition of lecithin promoted the formulation of liposomes, enhanced the solubility of resveratrol, and promoted its encapsulation [23].

### 3.5. Antioxidant Activity and UV Resistance

**Figure 4:**
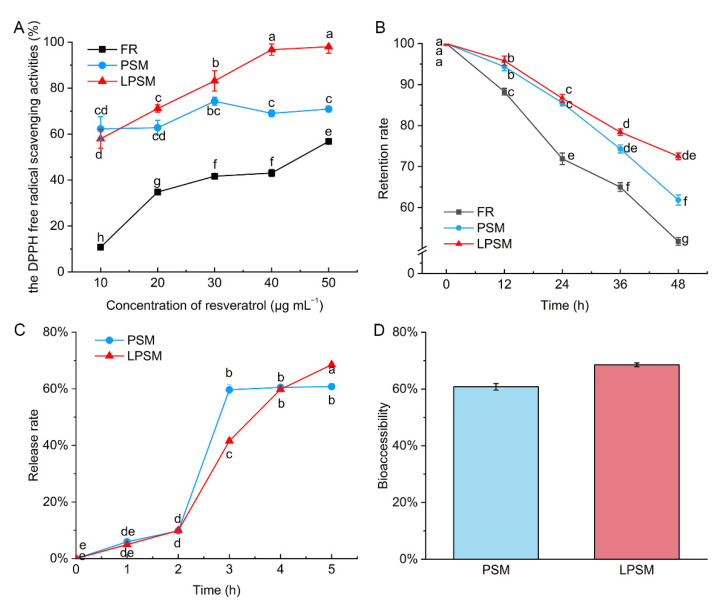
The antioxidant capability (**A**), retention rate (**B**) in response to ultraviolet, release rate in vitro (**C**) and bioaccessibility (**D**) of resveratrol in different forms. The first two hours were in simulated in gastric fluid, and the latter three hours in simulated intestinal fluid.FR, free resveratrol; PSM, polysaccharide self-assembled microspheres; LPSM, lecithin-polysaccharide self-assembled microspheres. Different letters indicate significant difference.

The DPPH radical scavenging method has been used to evaluate the antioxidant activity of various food systems [18]. To compare the difference between the antioxidant activity of free resveratrol and encapsulated resveratrol, DPPH free radical scavenging activities were determined among free resveratrol, PSM, and LPSM. Figure 4A shows an overview of the changes in antioxidant capability in the different forms. The DPPH free radical scavenging activities of free resveratrol increased with increased concentration. However, no good linear relationship was found between antioxidant capability and resveratrol concentration, which could result from the oxidation of free resveratrol. The DPPH free radical scavenging activities of PSM particles increased gradually with increased resveratrol concentration and rose to a maximum of 30 μg mL^−1^ and peaked at 74.27 ± 1.80%. The antioxidant capability of LPSM particles showed a steady increase with increased resveratrol concentration from 57.94 ± 4.19% at 10 μg mL^−1^ to 98.00 ± 2.82% at 50 μg mL^−1^ of resveratrol concentration. The resveratrol loaded in LPSM exhibited higher antioxidant activity compared to free resveratrol, indicating that the encapsulation of resveratrol by lecithin could enhance its stability. Similar results were found by Song et al. [26] in that chitosan coating elevated free radical scavenging activity. Increased antioxidant capacity by encapsulation may result from the many -OH and -NH_2_ groups in chitosan [27].

It is well-known that resveratrol is prone to chemical degradation in response to UV radiation [8,9]. In the present study, we found the encapsulation of PSM and LPSM improved the UV resistance of resveratrol. After exposure to UV for 48 h, LPSM exhibited the highest retention rate, at 72.50 ± 0.82%, followed by PSM at 61.83 ± 1.24%, and free resveratrol at 51.73 ± 0.94% (Figure 4B). The introduction of a lipid core into polysaccharide self-assembled microspheres significantly improved the stability of resveratrol under UV light.

### 3.6. Release of Resveratrol In Vitro

Upon sequential exposure in SGF and SIF, an increasing amount of resveratrol was detected in both PSM and LPSM (Figure 4C). During the time exposed to SGF, the recovered resveratrol increased at a low speed in both microspheres. At the end of digestion in SGF, the release rate in vitro of resveratrol in PSM was 9.79 ± 0.53%, and that of LPSM was 9.92 ± 0.3636%. These results suggest that the PSM and LPSM were relatively stable in SGF. When it came to SIF, the release of resveratrol in vitro exhibited completely different behaviors in PSM and LPSM. The recovered resveratrol from PSM increased sharply and reached 59.67 ± 1.76% after digestion in SIF for an hour, and afterwards was steady. However, the release rate in vitro from LPSM increased gradually, indicating a sustained release of resveratrol from LPSM in SIF. Finally, the resveratrol loaded in LPSM exhibited a higher bioaccessibility at 68.52 ± 0.68%. The slower release rate could be due to the presence of soybean lecithin, which requires an emulsification process during the degradation of LPSM in SIF. Leena et al. [14] applied an electrospinning approach to prepare resveratrol-loaded zein nanofibers for improved bioaccessibility of resveratrol. Only 33.8% of encapsulated resveratrol from the zein nanofibers was released in gastric conditions. When introduced to intestinal conditions, the resveratrol was released rapidly (up to 56.1% within 0.3 h). This release profile is similar to that of PSM in the present study. Vankayala et al. [28] studied the in-vitro release of free resveratrol and resveratrol-niosomes with non-ionic surfactants and fatty alcohol as stabilizers. The results showed the release rate was slowed by nano-encapsulation, but different rates were no observed between the gastric and intestinal conditions. Release behavior of resveratrol from the genipin cross-linked chitosan microsphere was observed [29]. The release of resveratrol was faster in an acidic medium owing to the faster swelling of chitosan and the physicochemical properties of resveratrol. The difference of resveratrol release from encapsulation in the studies mentioned above and the present study was probably caused by the degradability of the wall materials. The lipid core introduced to the polysaccharide self-assembled microspheres resulted in the inhibited release in SGF and the sustainable release in SIF. This approach for delivery could be used in other lipophilic nutraceuticals in the food industry, and to promote absorption in the intestine.

## 4. Conclusions

In the present study, lecithin-polysaccharide self-assembled microspheres (LPSM) were successfully developed for the encapsulation and delivery of resveratrol. Alginate and carboxymethyl chitosan were used in the formulation of multiple polysaccharide layer structures, and soybean lecithin was used as an inner layer of microspheres. Compared with PSM, the introduction of a lipid core in LPSM significantly improved the encapsulation efficiency of resveratrol and increased the zeta potential. The resveratrol loaded in LPSM exhibited elevated antioxidant capacity and improved stability under ultraviolet light. Both microspheres showed inhibited resveratrol release in simulated gastric digestion, while LPSM exhibited slower release of resveratrol in simulated intestinal fluid and had higher bioaccessibiliy. In conclusion, LPSM is a promising delivery system for resveratrol.

## Figures and Tables

**Table 1:** Particle size and Zeta potential of microspheres.

Sample	Particle Size (μm)	Zeta Potential (mV)	Encapsulation Efficiency (%)
PSM	6.422 ± 0.721 b	−21.18 ± 1.05 b	65.45 ± 0.43 b
LPSM	12.171 ± 0.960 a	−30.86 ± 1.37 a	92.78 ± 0.82 a

PSM, polysaccharide self-assembled microspheres; LPSM, lecithin-polysaccharide self-assembled microspheres. The different letters indicate significant differences.

## Data Availability

The data are contained within this article.

## Supplementary Materials

The following supporting information can be downloaded at: https://www.mdpi.com/article/10.3390/antiox11091666/s1, Figure S1: Fourier Transform Infrared (FTIR) spectra of polysaccharide self-assembled microspheres without resveratrol; Figure S2: Fourier Transform Infrared (FTIR) spectra of leci-thin-polysaccharide self-assembled microspheres without resveratrol; Figure S3: The High-performance liquid chromatography (HPLC) chromatogram of resveratrol standard; Figure S4: High-performance liquid chromatography (HPLC) chromatogram of polysaccharide self-assembled microspheres loaded resveratrol; Figure S5: High-performance liquid chroma-tography (HPLC) chromatogram of lecithin-polysaccharide self-assembled microspheres loaded resveratrol; Figure S6: High-performance liquid chromatography (HPLC) chromatogram of polysaccharide self-assembled microspheres without resveratrol; Figure S7: High-performance liquid chromatography (HPLC) chromatogram of lecithin-polysaccharide self-assembled mi-crospheres without resveratrol.
