# Supplementary material for: Lecithin-Polysaccharide Self-Assembled Microspheres for Resveratrol Delivery
Source: Antioxidants (Basel). 2022 Aug 26;11(9):1666. doi: 10.3390/antiox11091666 (PMC9495455; doi:10.3390/antiox11091666)
Supplement: Supplementary file 1 [file antioxidants-11-01666-s001.zip › antioxidants-1838559-supplementary.pdf]

Supplementary materials

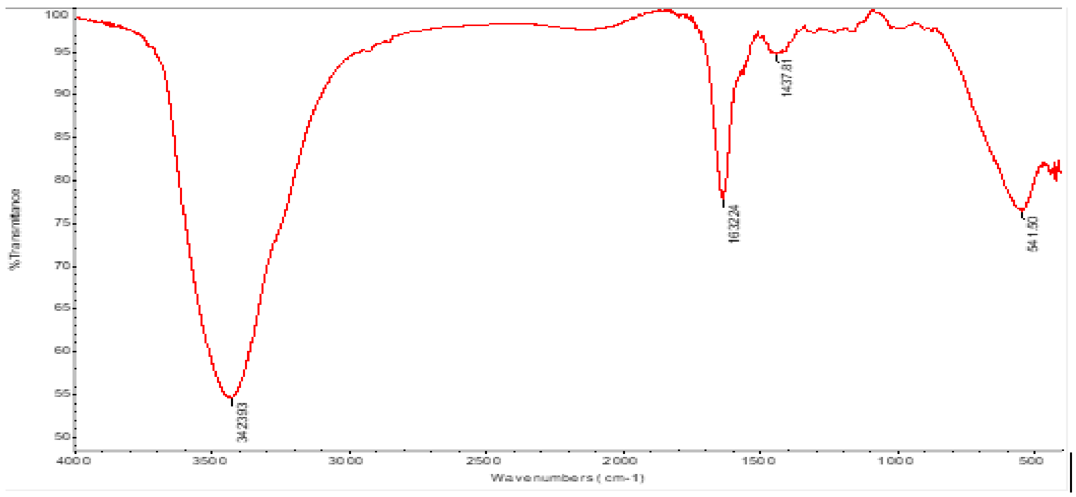

Figure S1. Fourier Transform Infrared (FTIR) spectra of polysaccharide self-assembled microspheres without resveratrol.

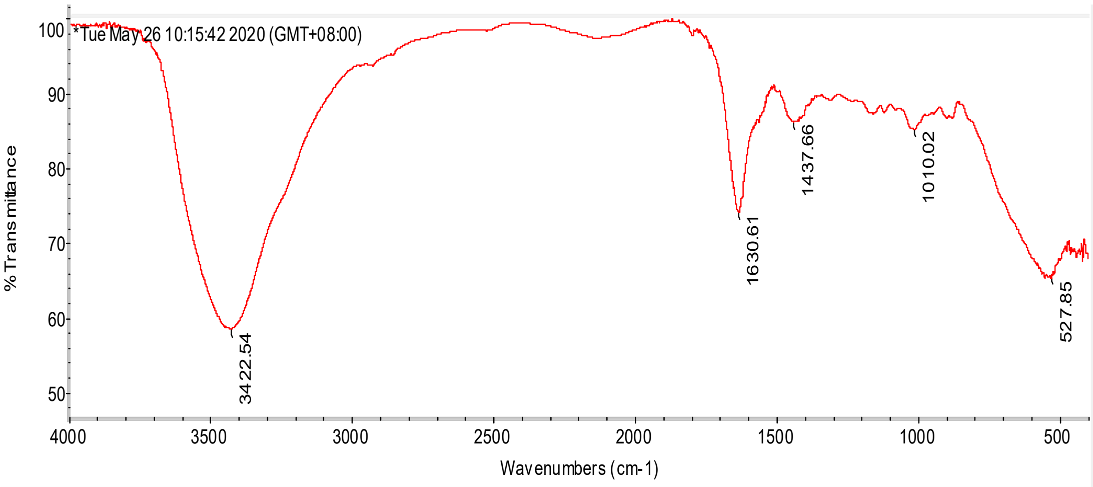

Figure S2. Fourier Transform Infrared (FTIR) spectra of lecithin-polysaccharide self-assembled microspheres without resveratrol

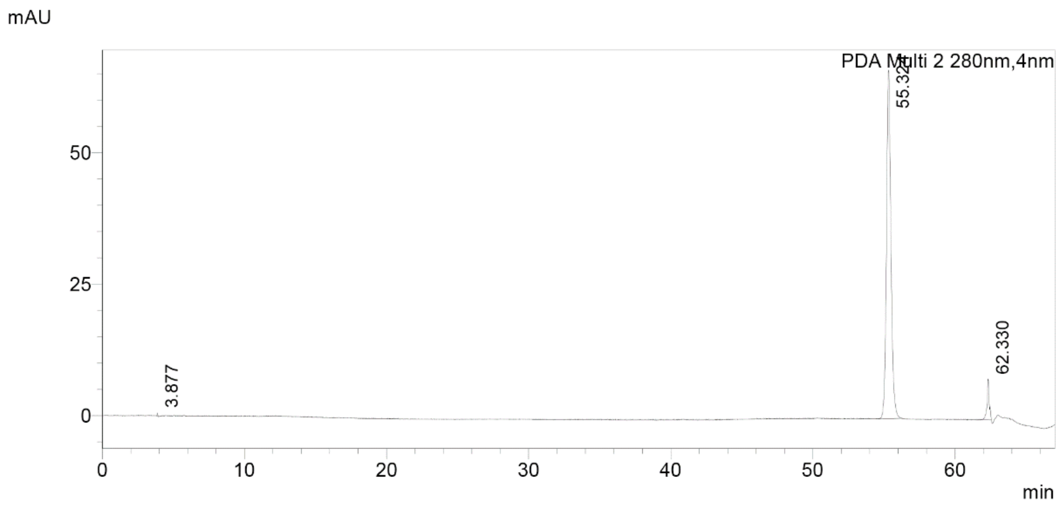

Figure S3. The High-performance liquid chromatography (HPLC) chromatogram of resveratrol standard.

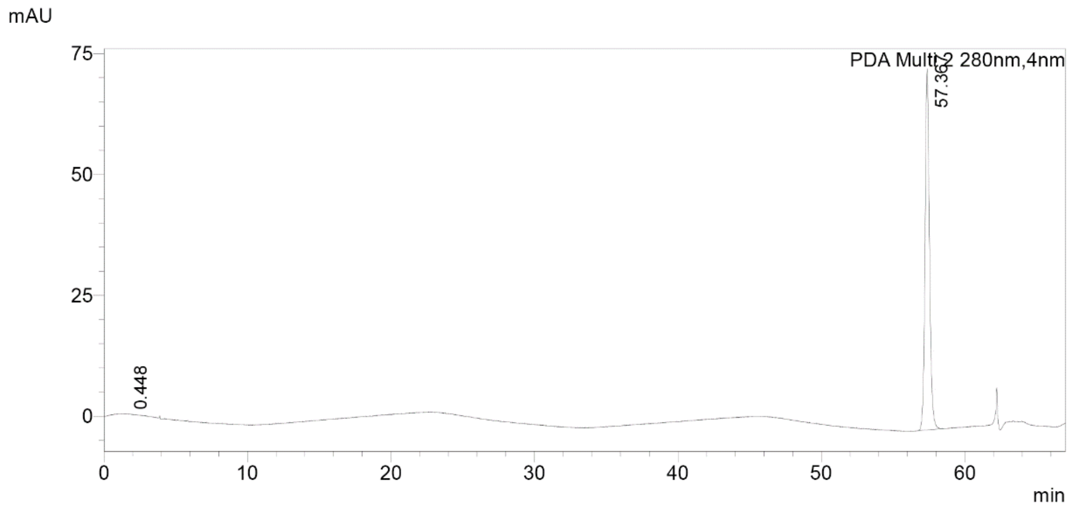

Figure S4. High-performance liquid chromatography (HPLC) chromatogram of polysaccharide self-assembled microspheres loaded resveratrol.

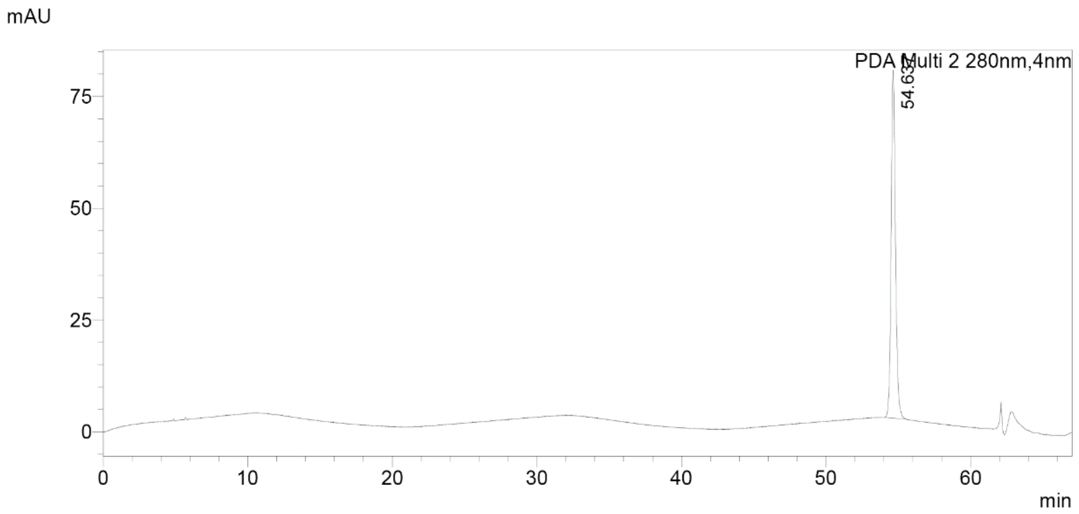

Figure S5. High-performance liquid chromatography (HPLC) chromatogram of lecithin-polysaccharide self-assembled microspheres loaded resveratrol

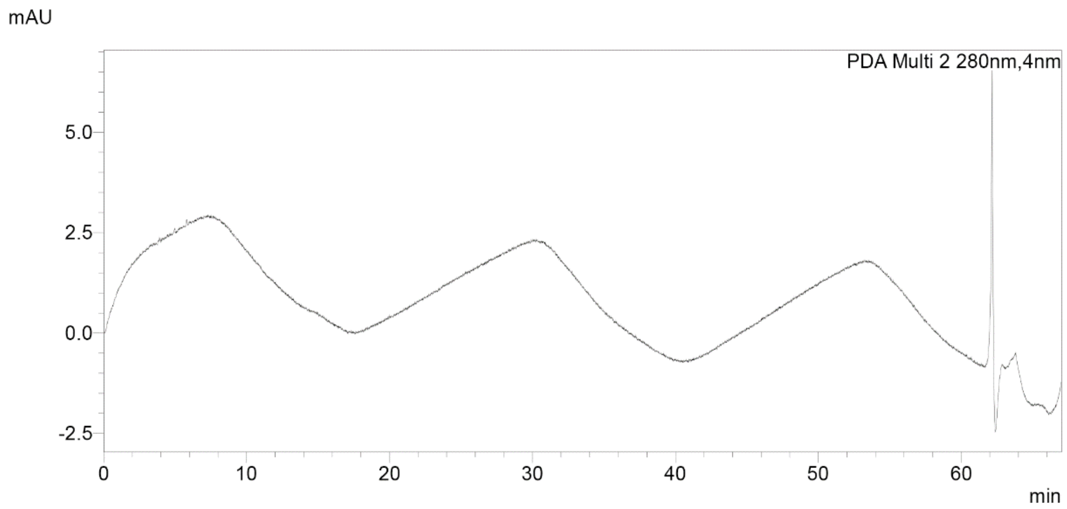

Figure S6. High-performance liquid chromatography (HPLC) chromatogram of polysaccharide self-assembled microspheres without resveratrol

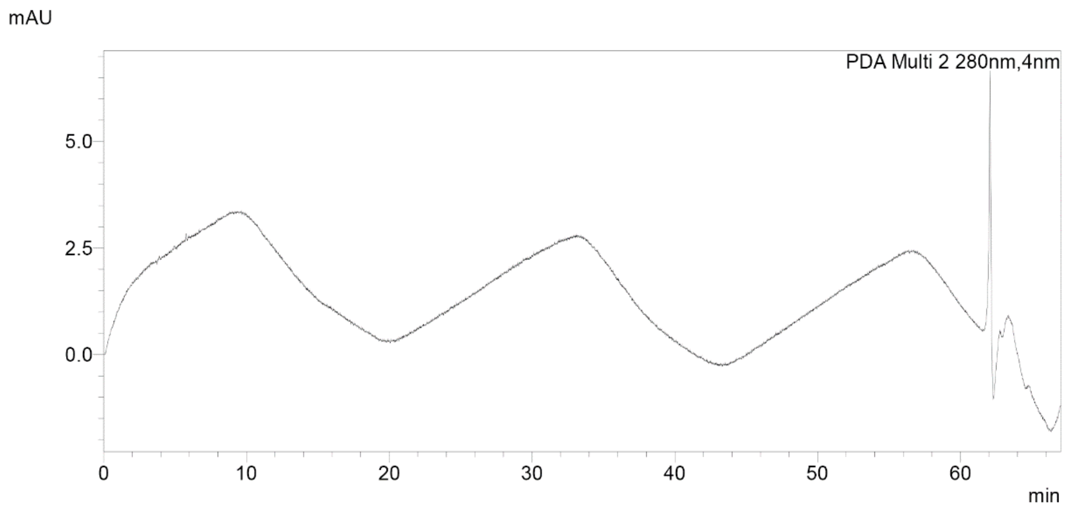

Figure S7. High-performance liquid chromatography (HPLC) chromatogram of lecithin-polysaccharide self-assembled microspheres without resveratrol.
